# Association between serum creatinine and 30 days all-cause mortality in critically ill patients with non-traumatic subarachnoid hemorrhage: analysis of the MIMIC-IV database

**DOI:** 10.3389/fneur.2024.1359749

**Published:** 2024-03-21

**Authors:** Yuan Zhong, Hao Sun, Wenjuan Jing, Lixian Liao, Jiayi Huang, Junqiang Ma, Weiqiang Chen

**Affiliations:** ^1^Department of Neurosurgery, The First Affiliated Hospital of Shantou University Medical College, Shantou, Guangdong, China; ^2^Department of Dermatology, The First Affiliated Hospital of Shantou University Medical College, Shantou, Guangdong, China; ^3^Department of Critical Care Medicine, Huizhou Third People’s Hospital, Guangzhou Medical University, Guangzhou, China; ^4^Department of Neurology, The First Affiliated Hospital of Shantou University Medical College, Shantou, Guangdong, China; ^5^Neuro-Intensive Care Unit, The First Affiliated Hospital of Shantou University Medical College, Shantou, Guangdong, China

**Keywords:** subarachnoid hemorrhage, intracranial aneurysm, serum creatinine, risk factor, stroke

## Abstract

**Background:**

Serum creatinine is a prognostic marker for various conditions, but its significance of spontaneous subarachnoid hemorrhage is still poorly understood. This study aims to elucidate the correlation between admission serum creatinine (sCr) levels and all-cause mortality within 30 days among individuals affected by non-traumatic subarachnoid hemorrhage (SAH).

**Methods:**

This cohort study included 672 non-traumatic SAH adults. It utilized data from the MIMIC-IV database from 2008 to 2019. The patients’ first-time serum creatinine was recorded. Subsequently, an examination of the 30-day all-cause mortality was conducted. Employing a multiple logistic regression model, a nomogram was constructed, while the association between sCr and 30-day all-cause mortality was evaluated using Kaplan–Meier survival curves. The calibration curve was employed to assess the model’s performance, while subgroup analysis was employed to examine the impact of additional complications and medication therapy on outcomes.

**Results:**

A total of 672 patients diagnosed with non-traumatic subarachnoid hemorrhage were included in the study. The mortality rate within this timeframe was found to be 24.7%. Multiple logistic regression analysis revealed that sCr served as an independent prognostic indicator for all-cause mortality within 30 days of admission for SAH patients [OR: 2(1.18–3.41); *p* = 0.01]. A comprehensive model was constructed, incorporating age, sCr, white blood cell count (WBC), glucose, anion gap, and partial thromboplastin time (PTT), resulting in a prediction model with an AUC value of 0.806 (95% CI: 0.768, 0.843), while the AUC for the test set is 0.821 (95% CI: 0.777–0.865).

**Conclusion:**

Creatinine emerges as a significant biomarker, closely associated with heightened in-hospital mortality in individuals suffering from SAH.

## Introduction

Non-traumatic subarachnoid hemorrhage (SAH) is a severe disease, mainly caused by the rupture of intracranial aneurysms, accounting for 2–7% of all strokes (1, 2). It is estimated that up to 40% of patients with non-traumatic SAH die in the hospital, resulting in disability and psychological disorders for the patients, and costing a massive amount of money and time (3, 4). Extensive clinical research and maximal treatment have been carried out, but SAH patients still show frustrating clinical outcomes (5). Therefore, there is an urgent need to establish non-invasive and cost-effective detection methods to help identify patients at increased risk of mortality and further optimize treatment.

Creatinine is related to the prognosis of various diseases, such as heart failure, coronary artery disease and cerebral hemorrhage transformation etc. (6–8). Creatinine is an important indicator of kidney function, and the elevation of creatinine levels may indicate kidney impairment (9). Acute kidney injury (AKI) is a common complication in critically ill patients and SAH patients, and its negative impact on outcomes is well known (10). Although the effect of sCr on the neurological function assessment of SAH patients has been described (11), the study’s sample size was small, and there was a lack of data on mortality in critically ill patients.

Therefore, we conducted a retrospective analysis through the MIMIC IV database to describe the association between sCr at admission and 30-day all-cause mortality.

## Methods

### Database introduction

The source of our data was the MIMIC-IV (v2.2), (Johnson, A, Bulgarelli, L, Pollard, T, Horng, S, Celi, LA, and Mark, R. MIMIC-IV (version 2.2). PhysioNet. (2022). doi: 10.13026/7vcr-e114a) large-scale, open-source database that was developed and maintained by the MIT Computational Physiology Laboratory. It included the records of all patients admitted to Beth Israel Deaconess Medical Center (BIDMC) from 2008 to 2019. The database offered comprehensive data for each patient, such as laboratory results, vital signs, medication administration, length of stay, etc. To protect patient privacy, all personal information was replaced with random codes and anonymized, so we did not need patient consent or ethical approval. The PhysioNet online platform allows downloading the MIMIC-IV (v2.2) database. One of the authors, Jiayi Huang, passed the exams on “Conflict of Interest” and “Data or Sample Only Research” (ID: 12408274) and completed the Collaborative Institutional Training Initiative (CITI) course to access the database. The research team then obtained the authorization to use the database and extract data. The main objective of the study is to construct a predictive model for mortality prediction.

### Population selection criteria

We performed a retrospective analysis of 672 non-traumatic SAH cases extracted from the online international database Medical Information Mart for Intensive Care (MIMIC-IV) between 2008 and 2019 based on the records of ICD-9 code 430 and ICD-10 codes I60, I600 to I6012, I6000 to I6002, I6020 to I6022, I6030 to I6032, and I6050 to I6052 (15). Patients who met the following criteria were included: (1) diagnosed with non-traumatic SAH at ICU admission; (2) aged ≥18 years; (3) first admission to ICU. Finally, 672 patients (289 males and 383 females) were enrolled and complete baseline data were collected.

### Data extraction

Creatinine, measured for the first time after admission to minimize the effect of subsequent treatments, was selected as the primary variable of interest. We also extracted potential confounders including demographics (age, sex), vital signs [heart rate, systolic and diastolic blood pressure, Mean arterial pressure (MAP), respiratory rate, and SpO2], comorbidities (myocardial infarction, peripheral vascular disease, peptic ulcer disease, liver disease, diabetes, paraplegia, cancer, and metastatic solid tumor), and laboratory tests (serum creatinine, white blood cells, neutrophils, lymphocytes, monocytes, serum glucose, anion gap, prothrombin time, international normalized ratio, and partial thromboplastin time). We used PostgreSQL software (v13.7.1) and Navicate Premium software (version 15) with structured query language (SQL) to perform data extraction. All the codes for computing demographic features, laboratory tests, comorbidities, and severity scores were obtained from the GitHub website (GitHub—MIT-LCP/mimic-iv: Deprecated. For the latest MIMIC-IV code see: https://github.com/MIT-LCP/mimic-code).

### Grouping and endpoint events

This study divided the patients into two subgroups based on their survival status in the hospital: those who lived for 30 days (*n* = 506) and those who passed away within 30 days (*n* = 166). Additionally, the dataset was divided into a training set and a validation set in a 7:3 ratio for statistical analysis, and the results are presented in the Supplementary material. The main outcome of interest was the all-cause mortality within 30 days of admission.

### Management of missing data

Variables with more than 15% missing values, such as monocytes, lymphocytes, neutrophils, basophils, eosinophils, albumin, GCS, albumin-globulin, total protein, and fibrinogen, were excluded to mitigate bias. For variables with less than 15% missing values (WBC, MCH, MCHC, MCV, RBC, RDW, heart rate, SBP, DBP, MBP, resp. rate, glucose, INR, PT, PTT, anion gap, sodium, bicarbonate, potassium, and creatinine), multiple imputation was applied to impute missing data using five replications and a chained equation approach. Rubin’s rules were then used for data pooling to combine the datasets, ensuring the robustness and reliability of the imputation results (12).

### Statistical analysis

Continuous variables were described using standard deviation (SD) ± mean or median interquartile range (IQR), while categorical variables were presented as percentages. Statistical differences between the survival and non-survival groups at 30 days were examined using Fisher’s exact, chi-square, or Kruskal-Wallis tests. T-tests or Wilcoxon rank-sum tests were used for continuous variables, and chi-square tests were used for categorical variables to compare differences between the two groups. Variables with *p* values <0.05 were included in the binary logistic regression model. Univariate (unadjusted) and multivariate (adjusted) binary logistic regression models were used to evaluate the relationship between clinical characteristics and patient prognosis, although this approach may overlook important variables and include non-significant variables. The final predictive model was constructed using variables with *p* values <0.05 in the multivariable logistic regression model. We conducted Cox regression analysis and present the results in the Supplementary material 1–3. The findings from both logistic regression and Cox regression methods are consistent. Column plots were then generated based on the logistic predictive model. Receiver operating characteristic (ROC) curve analysis was performed to evaluate the performance of the predictive model in the MIMIC-IV cohort. The area under the ROC curve (AUC) was calculated to summarize the diagnostic accuracy, sensitivity, and specificity. Additionally, a calibration curve was conducted to assess the clinical utility of the model. Kaplan–Meier curves were used to observe the relationship between creatinine and mortality rate in SAH patients. Creatinine was divided into two groups based on the baseline mean: the low serum creatinine group (creatinine <0.9 mg/dL) and the high serum creatinine group (creatinine ≥0.9 mg/dL). Subgroup analyses were conducted to assess the potential synergistic effect between elevated creatinine levels and complications or medication treatment in SAH patients, and a forest plot was generated to visualize the results. All statistical analyses were conducted using R 3.3.2 (http://www.R-project.org, The R Foundation) and Free Statistics software version 1.6 (Beijing, China).

## Results

### Baseline demographic and clinical characteristics

**Figure 1:**
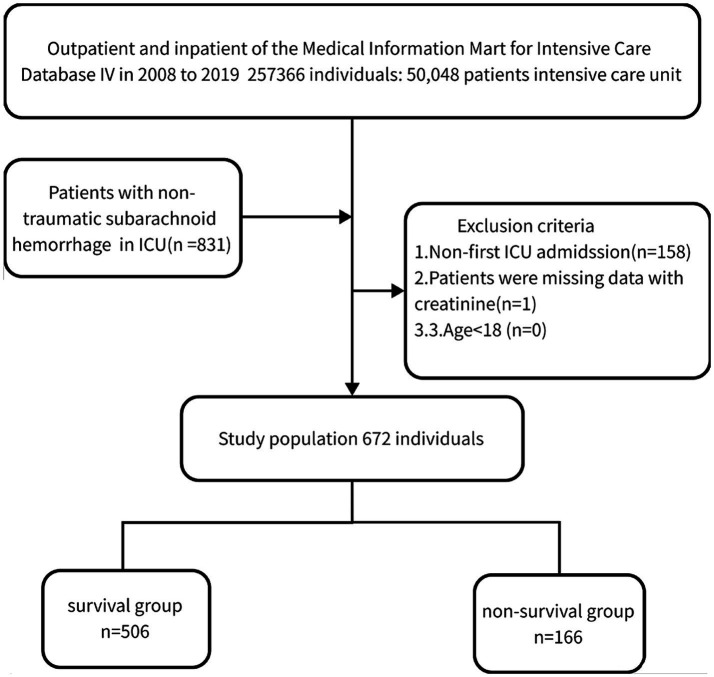
Flow of included patients.

Table 1 displays the baseline characteristics of the included study patients. Out of the 672 patients who fulfilled the inclusion criteria, as shown in Figure 1, 289 (43.0%) were male, and the median age was 61.6 (46.7, 76.5) years. The mortality rate within the 30-day timeframe amounted to 24.7%. Deceased individuals presented with an advanced age, augmented respiratory rate, elevated white blood cell count, increased red cell distribution width, heightened glucose levels, expanded anion gap, elevated international normalized ratio, prolonged prothrombin time, and activated partial thromboplastin time, along with elevated levels of creatinine (1.1 [0.1, 2.1] vs. 0.8 [0.4, 1.2], *p* < 0.001) in comparison to those who survived (all *p* < 0.05). The remaining covariates did not exhibit significant disparities between the two groups (*p* > 0.05).

**Table 1:** Differences between with favorable prognosis group and the unfavorable prognosis group in the development cohort.

Variables	Total (*n* = 672)	Yes (*n* = 506)	No (*n* = 166)	*p*
Gender				0.54
F	383 (57.0)	285 (56.3)	98 (59)	
M	289 (43.0)	221 (43.7)	68 (41)	
Admission age	61.6 ± 14.9	59.3 ± 14.3	68.6 ± 14.4	< 0.001
Heart rate	80.3 ± 17.8	79.8 ± 16.9	81.8 ± 20.4	0.222
Sbp	134.5 ± 23.4	134.8 ± 22.2	133.7 ± 27.0	0.601
Dbp	72.5 ± 16.5	72.7 ± 15.8	71.9 ± 18.6	0.611
Mbp	89.6 ± 19.2	89.6 ± 16.7	89.5 ± 25.5	0.982
Respi rate	17.9 ± 5.0	17.6 ± 4.9	18.9 ± 5.0	0.005
spo2	97.8 ± 3.1	97.8 ± 2.8	97.8 ± 3.9	0.980
Creatinine	0.9 ± 0.6	0.8 ± 0.4	1.1 ± 1.0	< 0.001
wbc	12.9 ± 5.8	12.5 ± 5.4	14.3 ± 6.8	< 0.001
Neutrophils	80.6 ± 12.7	80.5 ± 12.4	81.0 ± 14.0	0.720
Lymphocytes	13.3 ± 10.6	13.6 ± 10.5	12.3 ± 10.8	0.233
Monocytes	4.3 ± 2.4	4.3 ± 2.4	4.4 ± 2.2	0.853
Basophils	0.3 ± 0.3	0.4 ± 0.3	0.3 ± 0.3	0.042
Eosinophils	0.7 ± 1.1	0.7 ± 1.1	0.7 ± 0.8	0.504
mch	30.4 ± 2.4	30.4 ± 2.4	30.4 ± 2.4	0.944
mchc	33.6 ± 1.5	33.7 ± 1.5	33.4 ± 1.5	0.060
mcv	90.5 ± 6.2	90.4 ± 6.3	91.0 ± 6.1	0.237
rbc	4.3 ± 0.7	4.3 ± 0.6	4.2 ± 0.8	0.087
rdw	13.9 ± 1.7	13.7 ± 1.5	14.4 ± 1.9	< 0.001
Glucose	147.9 ± 52.2	139.4 ± 44.4	173.8 ± 64.4	< 0.001
Aniongap	15.7 ± 3.2	15.3 ± 2.9	17.1 ± 3.7	< 0.001
Sodium	138.9 ± 3.9	138.9 ± 3.5	138.9 ± 4.9	0.997
Potassium	4.0 ± 0.8	4.0 ± 0.8	4.1 ± 0.8	0.315
inr	1.2 ± 0.9	1.2 ± 0.6	1.4 ± 1.4	0.004
pt	13.5 ± 8.7	12.9 ± 6.4	15.1 ± 13.4	0.007
ptt	29.7 ± 15.0	28.8 ± 12.4	32.7 ± 20.9	0.004
Albumin	3.6 ± 0.6	3.6 ± 0.6	3.5 ± 0.6	0.166
Globulin	2.2 ± 0.7	2.5 ± 0.6	2.1 ± 0.8	0.303
Total protein	5.5 ± 1.2	6.0 ± 1.0	5.1 ± 1.3	0.165
Fibrinogen	347.3 ± 172.4	348.7 ± 170.1	345.2 ± 177.2	0.897
Myocardial infarct				0.971
0	623 (92.7)	471 (93.1)	152 (91.6)	
1	49 (7.3)	35 (6.9)	14 (8.4)	
congestive_heart_failure				0.717
0	619 (92.1)	469 (92.7)	150 (90.4)	
1	53 (7.9)	37 (7.3)	16 (9.6)	
peripheral_vascular_disease				0.729
0	615 (91.5)	463 (91.5)	152 (91.6)	
1	57 (8.5)	43 (8.5)	14 (8.4)	
cerebrovascular_disease				1
1	672 (100.0)	506 (100)	166 (100)	
Dementia, *n* (%)				0.372
0	665 (99.0)	503 (99.4)	162 (97.6)	
1	7 (1.0)	3 (0.6)	4 (2.4)	
chronic_pulmonary_disease				0.057
0	568 (84.5)	429 (84.8)	139 (83.7)	
1	104 (15.5)	77 (15.2)	27 (16.3)	
rheumatic_disease				0.309
0	661 (98.4)	499 (98.6)	162 (97.6)	
1	11 (1.6)	7 (1.4)	4 (2.4)	
peptic_ulcer_disease				0.64
0	666 (99.1)	502 (99.2)	164 (98.8)	
1	6 (0.9)	4 (0.8)	2 (1.2)	
mild_liver_disease				0.023
0	643 (95.7)	489 (96.6)	154 (92.8)	
1	29 (4.3)	17 (3.4)	12 (7.2)	
diabetes_without_cc				
0	597 (88.8)	455 (89.9)	142 (85.5)	0.198
1	75 (11.2)	51 (10.1)	24 (14.5)	
diabetes_with_cc				1
0	662 (98.5)	499 (98.6)	163 (98.2)	
1	10 (1.5)	7 (1.4)	3 (1.8)	
Paraplegia				0.634
0	590 (87.8)	448 (88.5)	142 (85.5)	
1	82 (12.2)	58 (11.5)	24 (14.5)	
renal_disease				0.966
0	636 (94.6)	487 (96.2)	149 (89.8)	
1	36 (5.4)	19 (3.8)	17 (10.2)	
malignant_cancer				0.471
0	650 (96.7)	491 (97)	159 (95.8)	
1	22 (3.3)	15 (3)	7 (4.2)	
severe_liver_disease				0.465
0	663 (98.7)	502 (99.2)	161 (97)	
1	9 (1.3)	4 (0.8)	5 (3)	
metastatic_solid_tumor				1
0	662 (98.5)	500 (98.8)	162 (97.6)	
1	10 (1.5)	6 (1.2)	4 (2.4)	
Aids				0.433
0	670 (99.7)	505 (99.8)	165 (99.4)	
1	2 (0.3)	1 (0.2)	1 (0.6)	

Baseline characteristics between survivors and non-survivors.

DBP, Diastolic blood pressure; SBP, Systolic blood pressure; MBP, Mean blood pressure: White blood cell; MCH, Mean corpuscular hemoglobin; MCHC, Mean corpuscular hemoglobin concentration; MCV, Mean corpuscular volume; RBC, Red blood cell; RDW, Red cell distribution width; INR, International normalized ratio; PT, Prothrombin time; PTT, Partial thromboplastin time.

### The sCr is an independent risk factor for all-cause mortality at 30 days of hospital admission

Unadjusted sCr was found to have a significant association with all-cause mortality within the first 30 days of hospitalization (odds ratio [OR]: 4.12, 95% confidence interval [CI]: 2.45–6.93, *p* < 0.001). In the multiple logistic regression analysis, sCr (adjusted OR: 1.94, 95% CI: 1.17–3.21, *p* = 0.01), age (adjusted OR: 1.05, 95% CI: 1.03–1.06, *p* < 0.001), WBC (adjusted OR: 1.05, 95% CI: 1.01–1.09, *p* < 0.009), glucose (adjusted OR: 1.01, 95% CI: 1–1.01, *p* < 0.001), anion gap (adjusted OR: 1.11, 95% CI: 1.04–1.19, p < 0.001), and PTT (adjusted OR: 1.02, 95% CI: 1–1.03, *p* = 0.021) were independent prognostic factors for in-hospital mortality in patients with spontaneous subarachnoid hemorrhage (SAH) after adjusting for confounding variables (Table 2).

**Table 2:** Multivariate regression analysis of factors associated with unfavorable outcome (mRS of 3–6) at 3 months.

Variable	OR_95CI	*p* value	adj.OR_95CI	adj.*p* value
Gender M	0.89 (0.63–1.28)	0.54		
Admission age	**1.05 (1.03–1.06)**	**<0.001**	**1.05 (1.03–1.06)**	**<0.001**
Heart rate	1.01 (1–1.02)	0.222		
Sbp	1 (0.99–1.01)	0.6		
Dbp	1 (0.99–1.01)	0.611		
Mbp	1 (0.99–1.01)	0.982		
Resp rate	1.05 (1.02–1.09)	**0.005**	1.03 (0.99–1.07)	0.202
Spo2	1 (0.94–1.06)	0.98		
Creatinine	**4.12 (2.45–6.93)**	**<0.001**	**1.94 (1.17–3.21)**	**0.01**
wbc	**1.05 (1.02–1.08)**	**0.001**	**1.05 (1.01–1.09)**	**0.009**
mch	1 (0.93–1.07)	0.944		
mchc	0.89 (0.8–1.01)	0.062		
mcv	1.02 (0.99–1.05)	0.238		
rbc	0.8 (0.62–1.03)	0.089		
rdw	1.26 (1.14–1.39)	**<0.001**	1.1 (0.98–1.25)	0.113
Glucose	**1.01 (1.01–1.02)**	**<0.001**	**1.01 (1–1.01)**	**<0.001**
Aniongap	**1.19 (1.13–1.26)**	**<0.001**	**1.11 (1.04–1.19)**	**0.001**
Sodium	1 (0.96–1.05)	0.997		
Potassium	1.11 (0.9–1.38)	0.33		
inr	1.33 (1.04–1.7)	**0.025**	2.88 (0.39–21.25)	0.299
pt	1.03 (1–1.05)	**0.035**	0.91 (0.75–1.1)	0.313
**ptt**	**1.01 (1–1.03)**	**0.006**	**1.02 (1–1.03)**	**0.021**

DBP, Diastolic blood pressure; SBP, Systolic blood pressure; MBP, Mean blood pressure: white blood cell; MCH, Mean corpuscular hemoglobin; MCHC, Mean corpuscular hemoglobin concentration; MCV, Mean corpuscular volume; RBC, Red blood cell; RDW, Red cell distribution width; INR, International normalized ratio; PT, Prothrombin time; and PTT, Partial thromboplastin time.

The bold values represent statistical significance, where any *p*-value less than 0.05 is indicated in bold to show that the result is statistically significant.

### ROC curve analysis and nomogram for predicting in-hospital mortality risk

**Figure 2:**
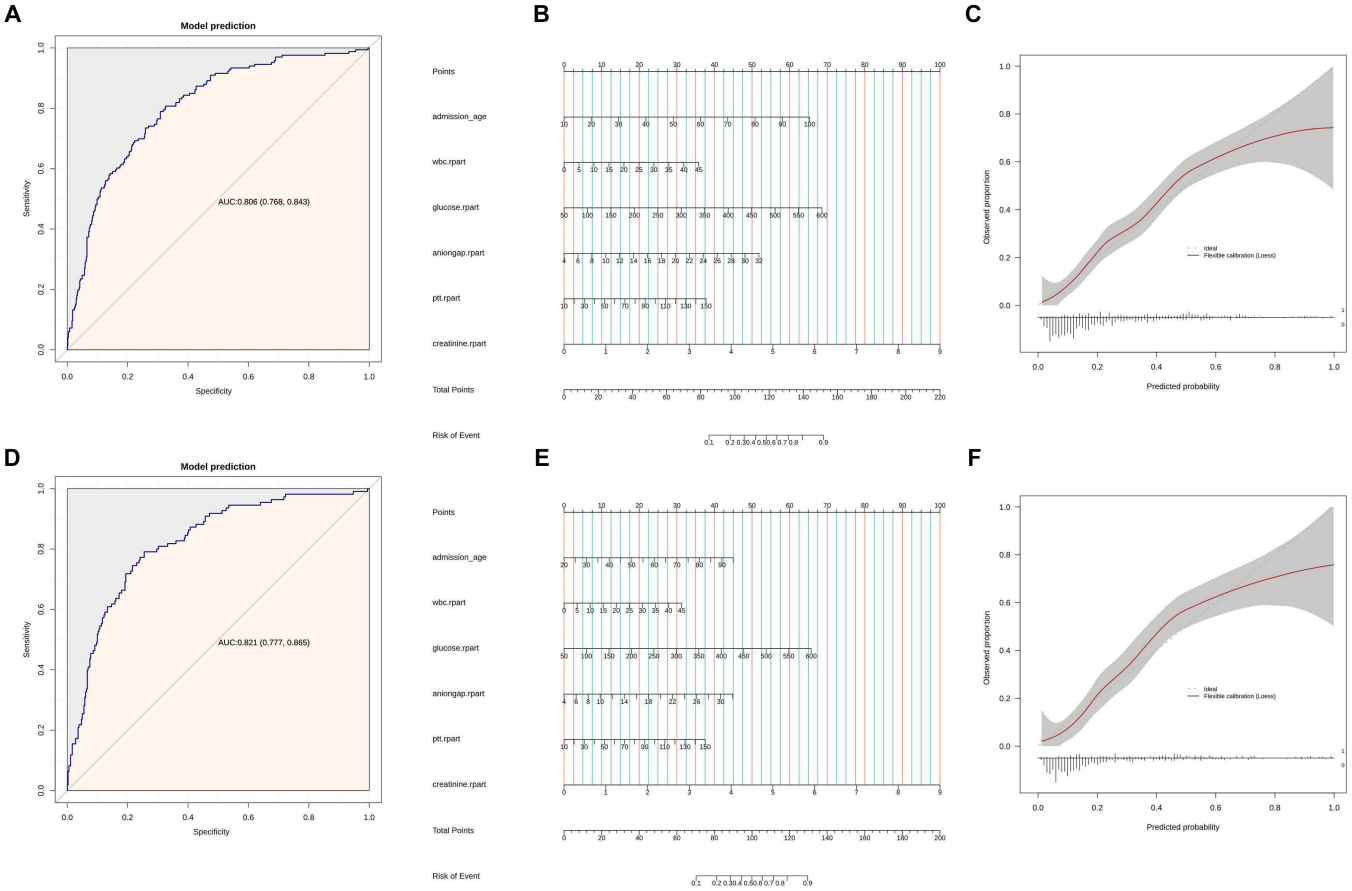
**(A)** ROC curves of model for predicting 30 days mortality. Model includes age, sCr, WBC, glucose, anion gap, and PTT. **(B)** Nomogram of Model includes age, sCr, wbc, glucose, anion gap, and PTT. **(C)** Calibration curve for the sCr. Shaded ribbons denoting 95% confidence intervals. Panels **(D–F)** are based on the test set data.

In Figures 2A,C, we used a multivariable logistic regression analysis to include the following variables: age, creatinine, WBC, glucose, anion gap, and PTT. We then plotted the ROC curves to assess the predictive ability of these variables for all-cause mortality within 30 days after admission of SAH patients. The area under the ROC curve was 0.806, with a 95% confidence interval of (0.768, 0.843). Moreover, the model exhibited a sensitivity of 80.72% and a specificity of 67.39%. The ROC curve for the test set was 0.821 (0.777, 0.865), with a sensitivity of 79.18% and a specificity of 62.89%. Based on the constructed model, nomogram was developed. Each patient would receive a total score based on the nomogram’s prognostic variables, corresponding to the predicted risk of in-hospital mortality (Figures 2B,E). A calibration curve was plotted to assess the consistency between the predicted probability of in-hospital mortality from the nomogram and the actual outcomes. As shown in Figures 2C,F, the calibration curve of the nomogram closely approximated the standard curve, indicating good consistency of the nomogram’s predictions (Figure 2D).

### Kaplan–Meier curve

**Figure 3:**
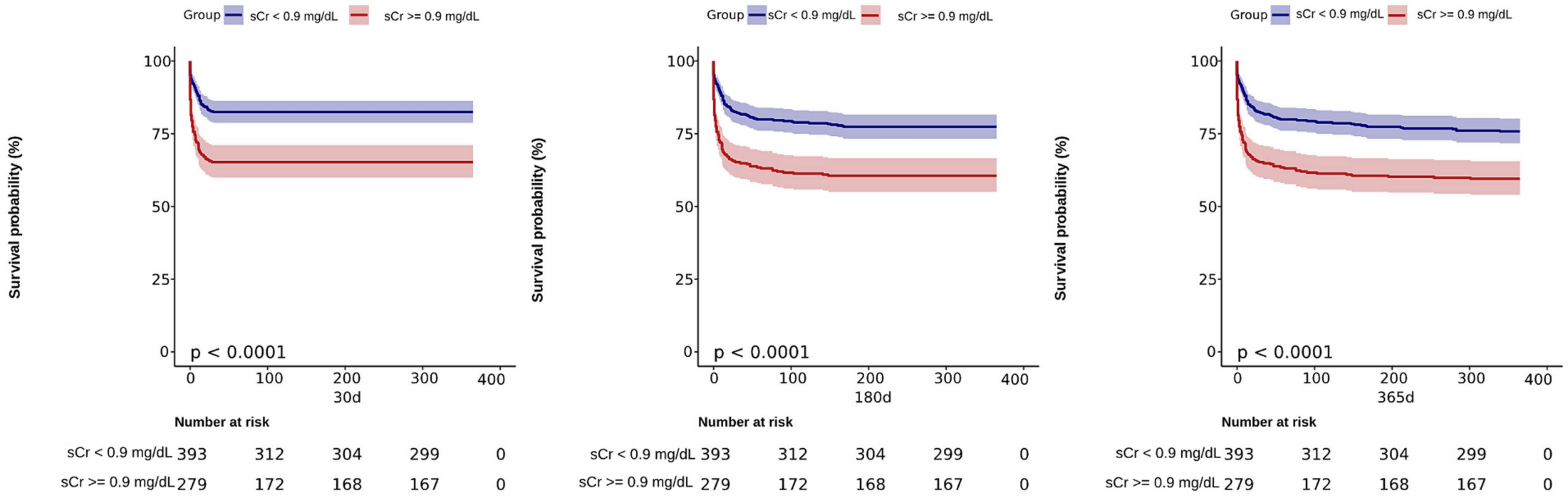
Kaplan–Meier survival analysis curves for 30, 180, and 365 days all-cause mortality. Creatinine 1: (sCr <0.9 mg/dL); Creatinine 2: (sCr ≥ 0.9 mg/dL).

Our study utilized an optimal threshold value to divide the SAH patients into two groups based on their creatinine levels. The high sCr group consisted of patients with sCr ≥ 0.9 (*n* = 166), while the low sCr group included patients with sCr < 0.9 (*n* = 506). To further evaluate the impact of sCr on patient outcomes, we conducted a Kaplan–Meier survival analysis and plotted the corresponding survival curves (Figure 3). The analysis revealed that the high sCr group had significantly higher mortality rates at 30, 180, and 365 days compared to the low sCr group (*p* < 0.001). This suggests that elevated sCr is associated with poorer prognosis and increased risk of mortality in SAH patients.

### Subgroup analysis and forest plots

**Figure 4:**
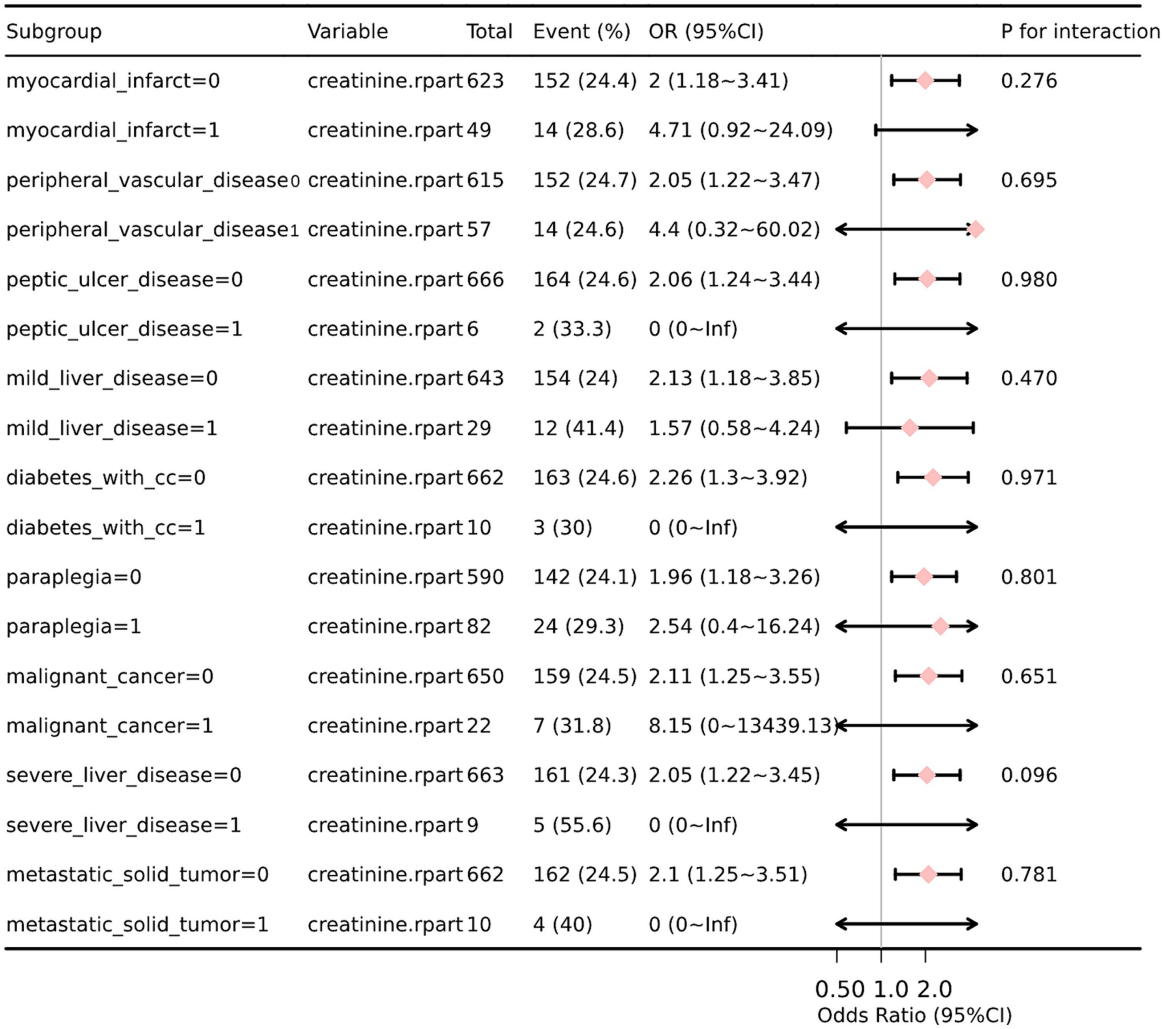
Forest plot for subgroup analysis of the relationship between 30 days mortality and sCr.

We performed additional stratification and interaction analyses to evaluate the relationship between sCr and the risk of in-hospital mortality in various subgroups, including myocardial infarction, peripheral vascular disease, peptic ulcer disease, liver disease, diabetes, paraplegia, cancer, and metastatic solid tumor. After adjusting for potential confounders such as age, WBC, glucose, anion gap, and partial thromboplastin time (PTT), the forest plot visually represented the results, demonstrating no significant interactions between creatinine and any of the subgroups (interaction *p* values ranging from 0.096 to 0.980) (Figure 4). These findings suggest that creatinine is an independent prognostic indicator for all-cause mortality in SAH patients. We conducted a subgroup analysis based on the use of antibiotics, CCB, alpha blockers, ARB, diuretics, ACEI, beta blockers, statins, 20% mannitol, and 25% albumin (Table 3). Only using CCB and 20% mannitol was associated with a lower mortality rate. We further analyzed the specific drugs within the CCB category (Table 4). We found that nimodipine significantly reduced the mortality rate in patients with elevated creatinine levels (OR: 0.3, 95% CI: 0.16–0.57, *p* < 0.001).

**Table 3:** Subgroup analysis of different medicine treatments.

Subgroup	n.total	n.event_%	OR_95CI	*p*_value
Creatinine<0.9				
Antibiotics 0	206	34 (16.5)	1(Ref)	
Antibiotics 1	186	35 (18.8)	1.17 (0.7–1.97)	0.549
Creatinine≥0.9				
Antibiotics 0	131	40 (30.5)	1(Ref)	
Antibiotics 1	121	30 (24.8)	0.75 (0.43–1.31)	0.31
Creatinine<0.9				
CCB 0	125	23 (18.4)	1(Ref)	
CCB 1	267	46 (17.2)	0.92 (0.53–1.6)	0.777
Creatinine≥0.9				
CCB 0	125	48 (38.4)	1(Ref)	
CCB 1	127	22 (17.3)	0.34 (0.19–0.6)	**<0.001**
Creatinine<0.9				
α Blocker 0	387	68 (17.6)	1(Ref)	
α Blocker 1	5	1 (20)	1.17 (0.13–10.66)	0.887
Creatinine<0.9				
α Blocker 0	248	69 (27.8)	1(Ref)	
α Blocker 1	4	1 (25)	0.86 (0.09–8.46)	0.901
Creatinine<0.9				
ARB 0	381	67 (17.6)	1(Ref)	
ARB 1	11	2 (18.2)	1.04 (0.22–4.93)	0.959
Creatinine≥0.9				
ARB 0	249	69 (27.7)	1(Ref)	
ARB 1	3	1 (33.3)	1.3 (0.12–14.62)	0.829
Creatinine<0.9				
Diuretics 0	272	44 (16.2)	1(Ref)	
Diuretics 1	120	25 (20.8)	1.36 (0.79–2.35)	0.266
Creatinine≥0.9				
Diuretics 0	177	48 (27.1)	1(Ref)	
Diuretics 1	75	22 (29.3)	1.12 (0.61–2.03)	0.72
Creatinine<0.9				
ACEI 0	378	65 (17.2)	1(Ref)	
ACEI 1	14	4 (28.6)	1.93 (0.59–6.33)	0.28
Creatinine≥0.9				
ACEI 0	246	68 (27.6)	1(Ref)	
ACEI 1	6	2 (33.3)	1.31 (0.23–7.31)	0.759
Creatinine<0.9				
β Blockers 0	231	43 (18.6)	1(Ref)	
β Blockers 1	161	26 (16.1)	0.84 (0.49–1.44)	0.529
Creatinine≥0.9				
β Blockers 0	157	48 (30.6)	1(Ref)	
β Blockers 1	95	22 (23.2)	0.68 (0.38–1.23)	0.204
Creatinine<0.9				
Statins 0	308	55 (17.9)	1(Ref)	
Statins 1	84	14 (16.7)	0.92 (0.48–1.75)	0.8
Creatinine≥0.9				
Statins 0	195	57 (29.2)	1(Ref)	
Statins 1	57	13 (22.8)	0.72 (0.36–1.43)	0.342
Creatinine<0.9				
20% mannitol	317	42 (13.2)	1(Ref)	
20% mannitol	75	27 (36)	3.68 (2.08–6.53)	**<0.001**
Creatinine≥0.9				
20% mannitol	215	49 (22.8)	1(Ref)	
20% mannitol	37	21 (56.8)	4.45 (2.16–9.17)	**<0.001**
Creatinine<0.9				
25% Albumin	369	65 (17.6)	1(Ref)	
25% Albumin	23	4 (17.4)	0.98 (0.32–2.99)	0.978
Creatinine≥0.9				
25% Albumin	237	67 (28.3)	1(Ref)	
25% Albumin	15	3 (20)	0.63 (0.17–2.32)	0.491

CCB, Calcium channel blockers; ARB, Angiotensin II receptor blockers; ACEI, Angiotensin converting enzyme inhibitors; 0 means the medicine is not used and 1 means the medicine is used.

The bold values represent statistical significance, where any *p*-value less than 0.05 is indicated in bold to show that the result is statistically significant.

**Table 4:** Subgroup analysis of different CCB medicine.

Subgroup	n.total	n.event_%	OR_95CI	*p*_value
Creatinine<0.9				
Verapamil 0	382	68 (17.8)	1(Ref)	
Verapamil 1	10	1 (10)	0.51 (0.06–4.12)	0.53
Creatinine≥0.9				
Verapamil 0	244	70 (28.7)	1(Ref)	
Verapamil 1	8	0 (0)	0 (0–Inf)	0.985
Creatinine<0.9				
Nimodipine 0	144	27 (18.8)	1(Ref)	
Nimodipine 1	248	42 (16.9)	0.88 (0.52–1.51)	0.649
Creatinine≥0.9				
Nimodipine 0	146	54 (37)	1(Ref)	
Nimodipine 1	106	16 (15.1)	0.3 (0.16–0.57)	**<0.001**

0 means the medicine is not used and 1 means the medicine is used.

The bold values represent statistical significance, where any *p*-value less than 0.05 is indicated in bold to show that the result is statistically significant.

## Discussion

In this study, we analyzed the association between sCr levels and 30-day mortality rate in SAH patients. This study only focused on establishing a predictive model and did not explore causal relationships. Our results indicate that higher sCr is associated with worse 30-day mortality outcomes in SAH patients. Multiple regression analysis demonstrated that after adjusting for other confounding factors, sCr is an independent risk factor for 30-day mortality in SAH patients. The novel line graph based on creatinine levels shows high predictive value.

Creatinine is an assessment indicator of kidney function and is a byproduct of muscle metabolism, primarily derived from the spontaneous non-enzymatic degradation of creatine phosphate (12). Creatine phosphate is stored in muscles, and its degradation produces creatinine, which is then excreted by the kidneys (13). Elevated creatinine levels may indicate impaired kidney function (14).

Research has demonstrated that chronic kidney disease (CKD) has an impact on the mortality rate of patients with aneurysmal subarachnoid hemorrhage (aSAH). One study found that patients with CKD had a higher risk of death during hospitalization for aSAH (15). A systematic review and meta-analysis revealed that CKD affects the mortality rate of patients with subarachnoid hemorrhage (SAH). The study found that ischemic stroke occurred at a higher relative frequency in CKD patients compared to hemorrhagic stroke (78.3 vs. 21.7%). However, as renal function declined, the relative frequency of hemorrhagic stroke gradually increased (16). These research findings suggest that chronic kidney disease may increase the risk of death in patients with aneurysmal subarachnoid hemorrhage. However, diagnosing CKD in patients with acute SAH poses relative difficulties (17). In a clinical setting, obtaining serum creatinine is convenient. We analyzed the first serum creatinine after admission to quickly assess the condition of SAH patients, avoiding the errors associated with repeated measurements and the influence of treatment on post-admission indicators.

Recently, creatinine has been widely used as a prognostic indicator for various critically ill patients. For example, some studies have found that elevated creatinine levels may be associated with the severity of coronary artery disease (6), acute pancreatitis (18), myocardial infarction (19), and the prognosis of symptomatic intracerebral hemorrhage following venous thrombolysis after acute ischemic stroke (8). However, there is limited research on the association between sCr and mortality rates in SAH patients. A previous study involving 369 SAH patients showed that patients with a sCr level ≥ 1.0 mg/dL had a higher likelihood of poorer prognosis (modified Rankin Scale score > 3) compared to SAH patients with sCr levels <1.0 mg/dL (11). These findings align with our study, which demonstrated that patients with sCr level ≥ 0.9 mg/dL had a higher mortality rate compared to those with sCr level < 0.9 mg/dL. Another study involving 66 patients with aSAH found that an early increase in the urea-to-creatinine ratio (UCR) after aneurysmal subarachnoid hemorrhage was independently associated with adverse clinical outcomes (*p* = 0.026) (20). However, compared to previous studies, our study has several differences. Firstly, we had a larger sample size, focusing on the non-traumatic SAH population in ICU. Secondly, the adjusted variables were also different. We adjusted for several well-known outcome parameters, disease severity, and complications of common critical illnesses.

Nimodipine, a calcium channel blocker, is widely used to reduce the occurrence of cerebral vasospasm and ischemic neurological dysfunction in patients with subarachnoid hemorrhage (SAH). Studies have demonstrated the effectiveness of nimodipine in improving patient prognosis and reducing adverse outcomes (21). Additionally, nimodipine has been shown to decrease the risk of cerebral vasospasm, delayed ischemic neurological dysfunction, and cerebral infarction in SAH patients (22). It is the only effective medication currently available for preventing post-SAH cerebral vasospasm (23). Our study found that the use of nimodipine significantly reduces the 30-day mortality rate in SAH patients with elevated creatinine levels. However, no significant impact was observed in patients with normal creatinine levels. We speculate that this may be due to the slower metabolism of nimodipine in patients with elevated creatinine, resulting in higher drug concentrations and influencing the outcome. Further prospective experiments may be necessary to investigate this hypothesis.

The administration of mannitol has been shown to potentially benefit in reducing intracranial pressure (ICP). Studies have demonstrated the effectiveness of mannitol in lowering ICP and its therapeutic efficacy in patients with subarachnoid hemorrhage (SAH) (24). Particularly, for patients unable to achieve ICP reduction, mannitol treatment has a positive impact on prognosis (25). In summary, mannitol may have a beneficial effect on reducing the mortality rate in SAH patients, although the specific outcomes may vary due to individual differences. Our study found that using mannitol reduces the 30-day mortality risk in patients, regardless of elevated creatinine levels. However, we did not observe a difference in the effectiveness of mannitol between patients with elevated creatinine levels, which suggests that mannitol’s potential renal toxicity does not affect its efficacy. Further research is needed to explore this phenomenon.

It is challenging to elucidate the exact mechanisms through which sCr in non-traumatic SAH patients is closely associated with overall mortality. However, several possible explanations can be proposed. One explanation is that creatinine elevation may be related to coronary artery atherosclerosis. We speculate that it may also be associated with cerebral vasculopathy, leading to increased vessel fragility and susceptibility to damage. Such effect could result in a higher risk of bleeding. Another explanation is that the increase in sCr may be a predictive factor for adverse neurological outcomes due to pre-existing renal dysfunction before SAH. Further research is needed to explore these hypotheses and elucidate the underlying mechanisms.

In summary, the relationship between sCr and 30-day mortality in SAH patients involves specific physiological and metabolic changes that require further research to explore the specific mechanisms. Overall, the factors mentioned above that affect sCr may increase the risk of adverse outcomes in SAH patients, but the exact mechanisms need further investigation.

## Limitation

Firstly, it is a retrospective cohort study with limitations such as potential measurement of missing data and data that may influence the results, such as mFisher grade, Hunt-Hess score, radiological data, and surgical information. Secondly, since creatinine data were only available at baseline upon ICU admission, this study can only establish the correlation between admission creatinine levels and hospital mortality, but not a causal relationship. The statistical methods we employed, such as univariate statistical tests, may lead to the risk of selecting insignificant variables while neglecting significant ones. Future studies may consider utilizing statistical techniques like Lasso regression to reduce statistical errors.

## Conclusion

Creatinine can serve as a critical parameter for predicting the prognosis of patients with spontaneous subarachnoid hemorrhage (SAH). The use of a line graph prediction model in conjunction with creatinine can accurately predict in-hospital mortality rates in SAH patients. Applying this prediction model can assist clinicians in assessing the patient’s condition and guide treatment decisions.

## Data availability statement

The data analyzed in this study were obtained from the Medical Information Mart for Intensive Care IV (MIMIC-IV) database; the following licenses/restrictions apply: to access the files, users must be credentialed users, complete the required training (CITI Data or Specimens Only Research), and sign the data use agreement for the project. Requests to access these datasets should be directed to PhysioNet, https://physionet.org/, DOI: 10.13026/6mm1-ek67.

## Ethics statement

Ethical approval was not required for the study involving humans in accordance with the local legislation and institutional requirements. Written informed consent to participate in this study was not required from the participants or the participants’ legal guardians/next of kin in accordance with the national legislation and the institutional requirements.

## Author contributions

YZ: Conceptualization, Data curation, Formal analysis, Funding acquisition, Investigation, Methodology, Project administration, Resources, Software, Supervision, Validation, Visualization, Writing – original draft, Writing – review & editing. HS: Conceptualization, Data curation, Formal analysis, Funding acquisition, Investigation, Methodology, Project administration, Resources, Software, Supervision, Validation, Visualization, Writing – review & editing. WJ: Conceptualization, Data curation, Formal analysis, Funding acquisition, Investigation, Methodology, Project administration, Resources, Software, Supervision, Validation, Visualization, Writing – review & editing. LL: Conceptualization, Data curation, Formal analysis, Funding acquisition, Investigation, Methodology, Project administration, Resources, Software, Supervision, Validation, Visualization, Writing – review & editing. JH: Conceptualization, Data curation, Formal analysis, Funding acquisition, Investigation, Methodology, Project administration, Resources, Software, Supervision, Validation, Visualization, Writing – review & editing. JM: Conceptualization, Data curation, Formal analysis, Funding acquisition, Investigation, Methodology, Project administration, Resources, Software, Supervision, Validation, Visualization, Writing – original draft, Writing – review & editing. WC: Conceptualization, Data curation, Formal analysis, Funding acquisition, Investigation, Methodology, Project administration, Resources, Software, Supervision, Validation, Visualization, Writing – original draft, Writing – review & editing.
