# Supplementary figures and images for: Association between serum creatinine and 30 days all-cause mortality in critically ill patients with non-traumatic subarachnoid hemorrhage: analysis of the MIMIC-IV database
Source: Front Neurol. 2024 Mar 21;15:1359749. doi: 10.3389/fneur.2024.1359749 (PMC10991831; doi:10.3389/fneur.2024.1359749)

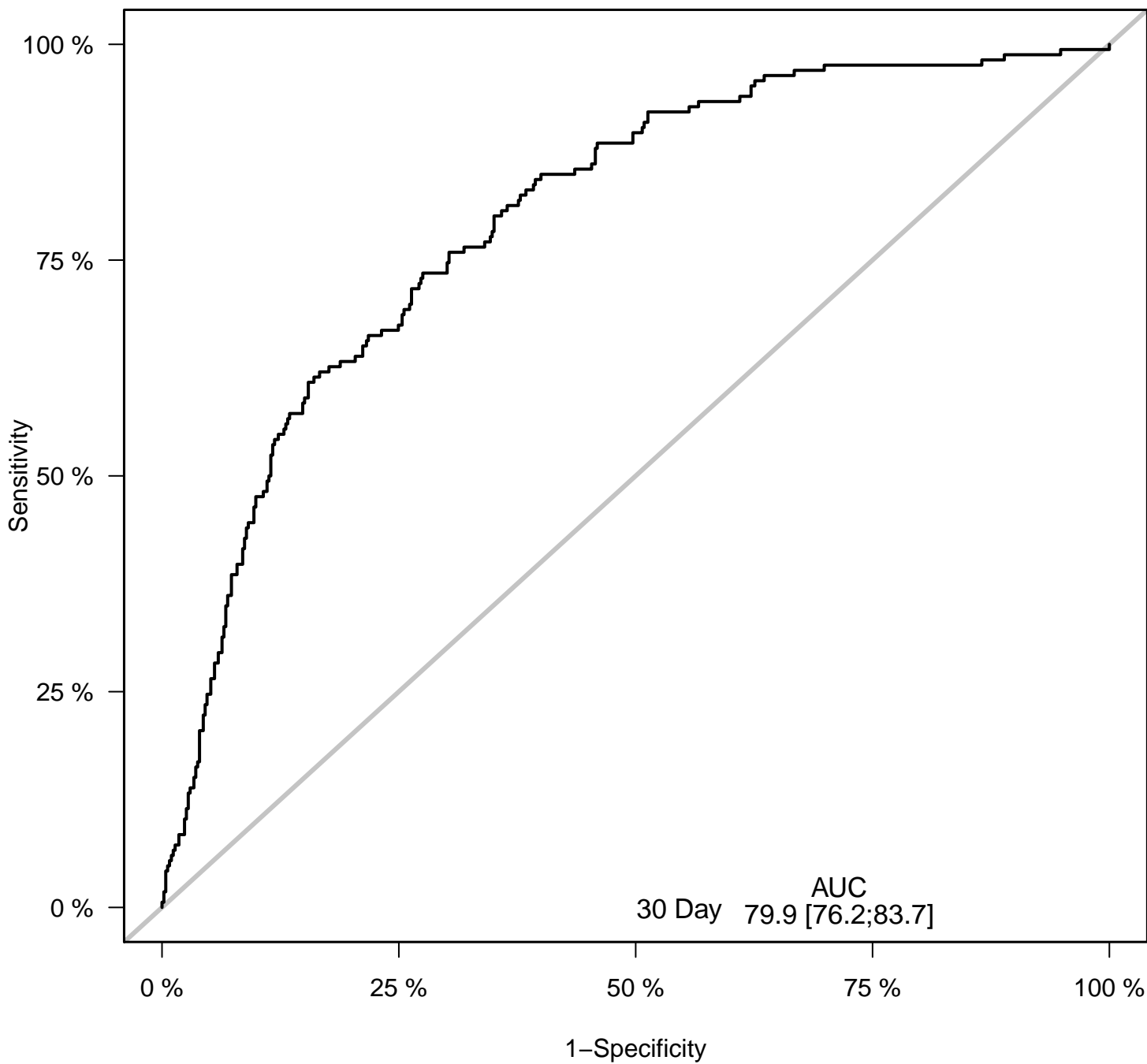

Supplement: Supplementary file 2 [file Data_Sheet_2.PDF]

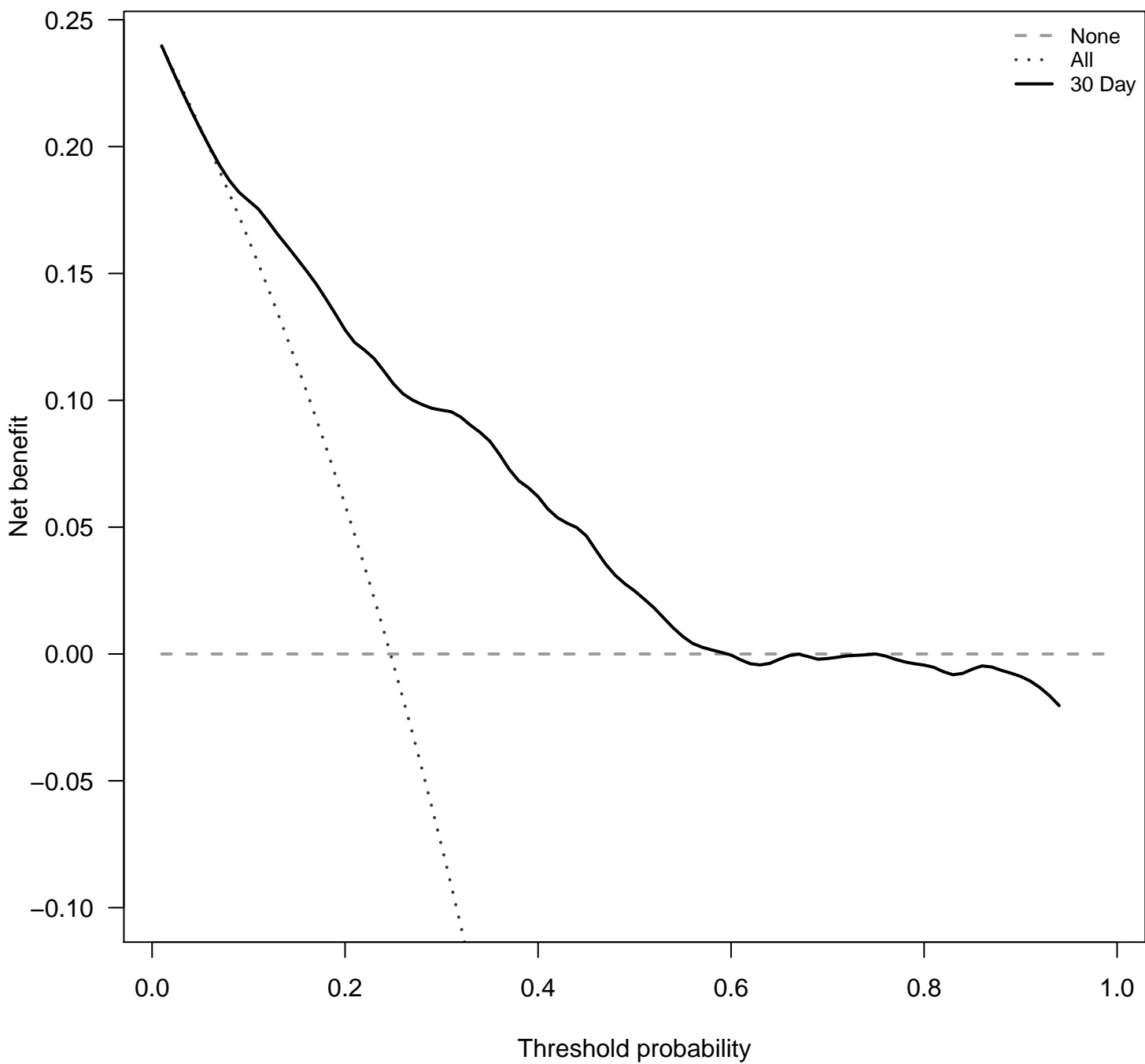

Supplement: Supplementary file 3 [file Data_Sheet_3.PDF]
